# Spread of Multidrug-Resistant Microorganisms

**DOI:** 10.3390/antibiotics11070832

**Published:** 2022-06-21

**Authors:** Silvia Di Lodovico, Teresa Fasciana, Mara Di Giulio, Luigina Cellini, Anna Giammanco, Gian Maria Rossolini, Alberto Antonelli

**Affiliations:** 1 Department of Pharmacy, University “G. d’Annunzio” Chieti-Pescara, 66100 Chieti, Italy; m.digiulio@unich.it (M.D.G.); l.cellini@unich.it (L.C.); 2 Department of Health Promotion, Mother and Child Care, Internal Medicine and Medical Specialties, University of Palermo, 90133 Palermo, Italy; teresa.fasciana@virgilio.it (T.F.); anna.giammanco@unipa.it (A.G.); 3 Department of Experimental and Clinical Medicine, University of Florence, 50134 Florence, Italy; gianmaria.rossolini@unifi.it (G.M.R.); albertoanton88@gmail.com (A.A.); 4 Clinical Microbiology and Virology Unit, Careggi University Hospital, 50134 Florence, Italy

Multidrug-resistant organisms (MDRO) are bacteria that exhibit acquired resistance to multiple antibiotics, reducing the efficacy of antimicrobial therapies. The spread of MDRO is currently one of the most important threats for public health, and it is associated with a remarkable burden in terms of increased morbidity, mortality, healthcare costs, and antibiotic use [1,2,3].

The extensive and, in most cases, inappropriate uses of antimicrobials, at both human and veterinary levels, contribute to the selective pressure at the basis of MDRO diffusion. Different factors contribute to the spread of resistant bacteria to humans and animals, including poorly prepared food, proximity, and inadequate hygiene. Resistant bacteria can be spread in the environment and food through contaminated wastewater or through wildlife. The best strategy to contrast the spread of MDRO is the implementation of a One Health approach, which involves the collaboration of multiple sectors to improve public health outcomes as defined by the World Health Organization [4].

This Special Issue includes a collection of ten research articles, communications, and reviews focused on the epidemiology and pathogenicity of MDRO. 

Alzahrani and coworkers investigated the role of backyard healthy chickens as a potential reservoir for MDR and virulent enterococci. In this study, antimicrobial resistance and virulence determinants, biofilm formation, plasmid content, and the clonality of *Enterococcus* spp isolates were investigated. Their results established the presence of nosocomial-associated clonal complex 17 and a variety of mobile genetic elements among the enterococci from backyard chickens, suggesting the possibility of their dissemination in backyard farms and related environments [5].

Animals, community, hospital, and industrial settings (including food-production) have been investigated as possible sources of Antimicrobial Resistance (AMR) by Massella and coworkers. Their article reaffirmed the role of food-producing animals as a reservoir of potential zoonotic pathogens, with variable antimicrobial and virulence traits among the sources investigated. In particular, rabbits and poultry represented the most concerning sources, carrying the highest number of antimicrobial resistance genes (ARGs) and virulence-associated genes. Poultry was associated with potential extraintestinal pathogenic *Escherichia coli* (ExPEC) lineages. Meanwhile, rabbits were a source of resistant and virulent *E. coli* pathogens with acquired colistin resistance determinant *mcr*-1 [6].

The role of animals in the spread of MDRO has also been described by Schmitt and coauthors, who reported extensive environmental contamination with extended spectrum β-lactamase-producing *Enterobacterales* (ESBL-E), carbapenemase-producing *Enterobacteriaceae* (CPE), and methicillin-resistant staphylococci (MRS) in a companion animal clinic in Switzerland. The study documented the plasmidic dissemination of *bla*_OXA-48_ in a companion animal clinic with low infection prevention and control (IPC) standards. This poses a worrisome threat to public health and highlights the need to foster standards in veterinary clinics to prevent the spread of MDRO into the community [7].

To reduce the circulation of MDRO, the programs of surveillance should be combined with the implementation of efficient disinfection protocols that employ various biocides. Geraldes and coworkers in the paper titled “Evaluation of a Biocide Used in the Biological Isolation and Containment Unit of a Veterinary Teaching Hospital” tested a recently developed biocide (Virkon™S), presenting a complex formulation (made by potassium peroxymonosulfate, sodium chloride, organic acids, an anionic surfactant, and an inorganic buffer) with mainly oxidative activity. Their results suggested that this novel biocide presented efficient antimicrobial activities against representatives of different bacterial species isolated from the Biological Isolation and Containment Unit (BICU). However, organic matter could interfere with its antimicrobial activity, and a slight change of antimicrobial susceptibility was observed in four enterococci, which is probably related to a general stress-induced response promoted by the sub-lethal levels of Virkon™ S [8].

The ability to produce biofilm and its correlation with antibiotic resistance was also reported by Fasciana and coauthors in their communication. The authors underlined the need to characterize the genetic diversity and biofilm formation properties of *Klebsiella pneumoniae* clinical isolates in order to help the control and management of associated infections and to limit their spread in the hospital environment. In addition, the characterization of the emerging high-risk clones can support the implementation of protective actions and infection control procedures [9].

A retrospective investigation from Southern Italy on the prevalence of Gram-negative bacteria (GNB) and their resistance in hospitalized patients by age, sex, and units from blood cultures (BCs) was conducted by Di Carlo and coworkers. The authors reported a higher prevalence of *K. pneumoniae* and *Acinetobacter baumannii* in the intensive care unit (ICU) and of *E. coli* in non-intensive care units (non-ICUs). The authors stressed the importance of the implementation of large community-level programs to prevent bacteremia caused by MDRO. The authors observed that local surveillance and the implementation of educational programs remain essential measures for slowing down the spread of resistance and, consequently, for increasing antibiotic lifespan [10].

Gentile and coworkers reported the importance of surveillance programs for nosocomial drug resistance. They suggested that whole-genome sequencing (WGS) proved to be a useful tool for elucidating the spreading dynamics of MDRO and can help in limiting their diffusion. According to the authors, the analysis of resistome and virulome by WGS, if available in real-time, could help to survey the alert nosocomial pathogens by directing the infection control team to focus its attention and resources in departments where surveillance is more necessary [11].

To reduce the diffusion of MDRO, the use of an appropriate antibiotic therapy is of paramount importance. Fiore and coworkers in a systematic review and meta-analysis titled “Ceftolozane-Tazobactam Combination Therapy Compared to Ceftolozane-Tazobactam Monotherapy for the Treatment of Severe Infections: A Systematic Review and Meta-Analysis” reported that combination therapy with ceftolozane-tazobactam (C/T), compared to C/T monotherapy, may reduce all-cause mortality in infections due to Gram-negative bacteria but did not increase the rate of clinical improvement [12].

The rate of multidrug resistance is also increasing in *Helicobacter pylori.* It is very important to have a good understanding of regional antibiotic resistance patterns of *H. pylori* for the implementation of effective empirical eradication therapies and adequate rescue therapies. The study conducted by Park and coworkers investigated the antibiotic resistance patterns of *H. pylori* and their impact on eradication in Seoul in recent years [13]. Their results suggested alarming data: the rate of multidrug resistance is increasing, and standard triple therapy (STT) is no longer an acceptable first-line option for *H. pylori* eradication in Korea.

The increasing spread of antibiotic-resistant *H. pylori* and the consequent limited therapeutic options available against this pathogen represent an important topic in gastroenterology. The aim of the review presented by Krzyżek and coworkers was to list compounds showing the ability to enhance the antimicrobial activity of classically used antibiotics against *H. pylori*. The antimicrobial properties, such as minimal inhibitory concentrations and minimal bactericidal concentrations, dose- and time-dependent mode of action, and, if characterized, anti-biofilm and/or in vivo activity of these compounds are also reviewed [14]. 

In conclusion, this Special Issue collected articles and reviews that underlined the need for robust surveillance systems that are essential for combating antibiotic resistance at both hospital and environmental levels, emphasizing the necessity of an “One Health” approach. The importance of both phenotypic and genotypic approaches for the characterization of MDRO, and the necessity of novel effective antimicrobials and biocides active against MDRO was also emphasized.
